# Cathelicidins PMAP-36, LL-37 and CATH-2 are similar peptides with different modes of action

**DOI:** 10.1038/s41598-019-41246-6

**Published:** 2019-03-18

**Authors:** Maaike R. Scheenstra, Matthias van den Belt, Johanna L. M. Tjeerdsma-van Bokhoven, Viktoria A. F. Schneider, Soledad R. Ordonez, Albert van Dijk, Edwin J. A. Veldhuizen, Henk P. Haagsman

**Affiliations:** Department of Infectious Diseases and Immunology, Division of Molecular Host Defence, Faculty of Veterinary Medicine, Utrecht University, Utrecht, The Netherlands

## Abstract

Host defense peptides (HDPs) play a pivotal role in innate immunity and have, in addition to antimicrobial activity, also important immunomodulatory functions. Bacteria are less likely to develop resistance against HDPs because these peptides target and kill bacteria in multiple ways, as well as modulate the immune system. Therefore, HDPs, and derivatives thereof, are promising alternatives to traditional antibiotics. Hardly anything is known about the immunomodulatory functions of porcine cathelicidin PMAP-36. In this study, we aimed to determine both antibacterial and immunomodulatory activities of PMAP-36 comparing the properties of PMAP-36 analogs with two well-studied peptides, human LL-37 and chicken CATH-2. Transmission electron microscopy revealed different killing mechanisms of *E*. *coli* for PMAP-36, CATH-2 and LL-37. LL-37 binds LPS very weakly in contrast to PMAP-36, but it inhibits LPS activation of macrophages the strongest. The first 11 amino acids of the N-terminal side of PMAP-36 are dispensable for *E*. *coli* killing, LPS-neutralization and binding. Deletion of four additional amino acids resulted in a strong decrease in activity. The activity of full length PMAP-36 was not affected by monomerization, whereas the shorter analogs require dimerization for proper immunomodulatory activity but not for their antibacterial activity.

## Introduction

Cathelicidins constitute a family of host defense peptides (HDPs) and play an important role during the innate immune response^1^. They consist of a highly conserved N-terminal region, containing a signal peptide and a cathelin domain, while the C-terminal region represents the highly variable domain of the active peptide^2–4^. Although the sequences of cathelicidins are highly variable, almost all cathelicidins show, in simple media, direct antimicrobial activity against many different bacteria^3,5–7^, viruses^8–10^, fungi^11^, and parasites^12,13^.

Besides their direct antimicrobial activities, cathelicidins can also modulate the immune response. These peptides can induce chemotaxis directly or indirectly by inducing chemokine release^3,14,15^. In addition, cathelicidins have been shown to be involved in phagocytosis^3,16–18^, neutralization of LPS or LTA during TLR stimulation^3,15,19,20^ or enhancement of DNA uptake and subsequent TLR-9 activation^3,5,21,22^ and they can skew macrophage differentiation towards a pro-inflammatory phenotype^23^. Bacteria are less likely to develop resistance against HDPs because they do not only target and kill bacteria in multiple ways but also modulate the immune system. This dual function makes HDPs promising alternatives to antibiotics.

In humans only one cathelicidin has been identified, LL-37^2^, while in chicken four cathelicidins have been identified, CATH-B1 and CATH-1-3^24^. The pig has an even larger arsenal of 11 cathelicidins: protegrin-1-5, prophenin-1-2, and pig myeloid antibacterial peptide (PMAP)-23, -36, and -37^4^. Human LL-37 is a 37 amino acid cationic (6+) peptide and has been widely studied. LL-37 penetrates the bacterial membrane and forms pores in the membrane. LL-37 adopts an α-helical structure^25^, which resembles the structure of PMAP-36. In addition, many different immunomodulatory functions have been described for LL-37^26–28^. Chicken CATH-2 is a 26 amino acid cationic (11+) arginine-lysine-rich peptide, consisting of two α-helical regions with a proline induced hinge region^29^. CATH-2 displays strong antimicrobial activities against many different pathogens, e.g. Gram-positive and Gram-negative bacteria^29–32^ and fungi^32,33^. In addition, CATH-2 has been shown to have immunomodulatory capacities^29,30,34^.

Porcine cathelicidin PMAP-36 was first described in 1994 as a 36-amino acids long, highly cationic (13+), amphipathic, α-helical antimicrobial peptide with a proline-induced hinge region. Most of its cationic residues are located in the first 20 amino acids or the active peptide, forming an α-helical structure^35^. The C-terminal end of the active peptide is proline rich and contains most of the hydrophobic amino acids. In addition, the 35^th^ amino acid is a cysteine residue, enabling dimerization of PMAP-36 by intermolecular S-S bridging, resulting in a net charge of 26+^36^. PMAP-36 is stored in the primary granules of neutrophils and secreted after phorbol 12-myristate 13-acetate (PMA) stimulation^37^. Strong antimicrobial activity was found against both Gram-negative and Gram-positive bacteria^35,36,38,39^, as well as Candida^39^. The N-terminal part of PMAP-36 is not required for direct killing^38,39^. In addition, PMAP-36 was found to modulate the TLR response by strongly neutralizing LPS- and LTA-mediated activation of macrophages by TLR-4 and TLR-2, respectively^3^. On the other hand, the peptide strongly enhances bacterial DNA (TLR-9) stimulation of porcine dendritic cells (pDC), resulting in an increased IFN-α response^21^. This effect appears to be cell or species specific, since no enhanced activation of murine macrophages by DNA stimulation was found^3^.

In this study, the antibacterial and immunomodulatory properties of PMAP-36 were studied in a systematic manner. By designing truncated analogs, the importance of the first 15 N-terminal amino acids for the biological activities was determined. We showed that the first 11 amino acids were dispensable for the peptide function whereas deletion of 4 additional amino acids causes a complete loss of function. In addition, the effect of dimerization was studied, by designing analogs that were unable to form dimers. Prevention of dimerization slightly enhanced the antimicrobial activity and reduced the cytotoxic effects.

## Material and Methods

### Reagents

CATH-2, PMAP-36 and its analogs were synthesized by Fmoc-chemistry at China Peptides (CPC scientific, Sunnyvale, CA, USA). LL-37 was synthesized by Fmoc-chemistry at the Academic Centre for Dentistry Amsterdam (Amsterdam, the Netherlands). All peptides were purified by reverse phase high-performance liquid chromatography to a purity of >95%. Sequences of the peptides used in this study are presented in Table 1.

**Table 1:** Peptides used.

Peptide	Short name	Sequence*	Length	Charge
LL-37	LL-37	LLGDFFRKSKEKIGKEFKRIVQRIKDFLRNLVPRTES	37	6+
CATH-2	CATH-2	RFGRFLRKIRRFRPKVTITIQGSARF-NH_2_	26	8+
PMAP-36	PMAP-36	Ac-GRFRRLRKKTRKRLKKIGKVLKWIPPIVGSIPLGCG	36	13+
PMAP(7–36)	P7	Ac-RKKTRKRLKKIGKVLKWIPPIVGSIPLGCG	30	10+
PMAP(12–36)	P12	Ac-KRLKKIGKVLKWIPPIVGSIPLGCG	25	6+
PMAP(16–36)	P16	Ac-KIGKVLKWIPPIVGSIPLGCG	21	3+
PMAP(1–33) monomer	mP1	Ac-GRFRRLRKKTRKRLKKIGKVLKWIPPIVGSIPLGS	33	13+
PMAP(7–33) monomer	mP7	Ac-RKKTRKRLKKIGKVLKWIPPIVGSIPLGS	27	10+
PMAP(12–33) monomer	mP12	Ac-KRLKKIGKVLKWIPPIVGSIPLGS	22	6+
PMAP(16–33) monomer	mP16	Ac-KIGKVLKWIPPIVGSIPLGS	18	3+

### Mammalian cell lines and primary cell isolation

The murine RAW264.7 macrophage cell line (ATCC-TIB-71) (ATCC, Manassas, VA, USA) was cultured in high glucose containing DMEM (Thermo Fisher Scientific, Waltham, MA, USA) supplemented with 10% FCS (Bodinco B.V., Alkmaar, the Netherlands) at 37 °C in 5.0% CO_2_. The porcine 3D4/31 macrophage cell line (ATCC-CRL-2844) (ATCC) was cultured in RPMI-1640 (Thermo Fisher Scientific), supplemented with 1% non-essential amino acids (Thermo Fisher Scientific) and 10% FCS (Bodinco B.V.) at 37 °C in 5.0% CO_2_.

Red blood cells (RBCs), peripheral blood mononuclear cells (PBMCs) and granulocytes were isolated from freshly isolated porcine blood. All animals were kept under the guidelines and approval of the animal ethical committee of Utrecht University in the Netherlands.

RBCs were isolated by centrifugation of whole blood at 220xg for 15 minutes, after which the platelet rich plasma and buffy coat were removed. The RBCs were washed three times with PBS and centrifuged at 1360xg for 10 minutes each time to remove all granulocytes.

PBMCs and granulocytes were isolated by Ficoll-Paque Plus separation (ρ = 1.007 g/ml; GE Healthcare Bio-Science AB, Sweden). PBMCs formed the buffy ring on the Ficoll-Paque Plus and were washed once before resuspension in RPMI-1640 supplemented with 10% FCS and 1% Pen/Strep. For isolation of the granulocyte fraction, RBCs were lysed with an isotonic ammonium chloride buffer (155 mM NH_4_Cl, 10 mM KHCO_3_, 0.1 mM EDTA) for 5–10 minutes on ice, washed 1x with PBS and were resuspended in RPMI-1640 supplemented with 10% FCS and 1% Pen/Strep. Both PBMCs and granulocytes were kept at 37 °C in 5.0% CO_2_ during the assays.

### Colony count assay

*Escherichia coli* O78 (*E*. *coli*) (Clinical isolate, Zoetis Animal Health, Kalamazoo, MI, USA) was grown in Mueller Hinton Broth (MHB) (Becton Dickinson, USA) to mid-logarithmic phase under constant shaking (200 RPM) at 37 °C. Antimicrobial activity of the peptides was tested using the colony count assay as described before^40^. In short, 10^6^ CFU/ml *E*. *coli* were incubated with different concentrations peptides (0.63–40 μM) for 3 h at 37 °C. Next, 10-fold dilutions were made, of which 100 μl of the dilutions was spread to tryptic soy agar (TSA) (Oxoid, Basingstoke, UK) plates. Plates were incubated overnight at 37 °C and colonies were counted to determine the number of surviving bacteria. Minimal Bactericidal Concentration (MBC) was defined as ≤100 CFU/ml, the detection limit of the assay.

### Transmission electron microscopy

For transmission electron microscopy (TEM), *E*. *coli* was used in a higher concentration, (5*10^8^ CFU/ml). The MBC value was determined for this higher density of bacteria using the colony count assay (MBCs: PMAP-36–20 μM, LL-37–160 μM, CATH-2–40 μM). *E*. *coli* was incubated with the peptides at MBC concentration or sub-MBC concentration (4x below MBC concentration). After 60 min at 37 °C, fixative (4% glutaraldehyde, 5 mM CaCl_2_, 10 mM MgCl_2_ (all from Merck, Darmstadt, Germany) in 0.1 M sodium cacodylate buffer (Sigma) pH 7.4) was added 1:1 to the bacterial-peptide mixture and left for fixation overnight at 4 °C. Samples were washed three times in 0.1 M sodium cacodylate buffer and resuspended in 4% low melting point agarose (Sigma). Samples were post-fixed with 1% osmium tetroxide (Electron Microscopy Sciences (EMS), Hatfield, PA, USA), 1.5% K_3_Fe(CN)_6_ (Merck) and 65 mM sodium cacodylate buffer for 2 hours at 4 °C to improve the contrast. After extensive washing with distilled water (5 × 10 min), samples were incubated for 60 min with 0.5% uranyl acetate (EMS) at 4 °C to improve cytoplasmic contrast. After washing with distilled water (3 × 10 min), samples were dehydrated with increasing concentration ethanol and embedded in Epon. Ultrathin sections (50 nm) were made with the Leica UCT ultramicrotome (Leica, Vienna, Austria), stained with uranyl acetate and lead citrate on an AC20 system (Leica) and pictures were taken at 80 kV with the FEI Tecnai 12 electron microscope (FEI, Eindhoven, the Netherlands). Morphological effects were quantified for around 100 cells per condition.

### Cell viability

RAW264.7 or 3D4/31 cells (5*10^4^ cells/well) were seeded in a 96-well tissue culture treated microtiter plate and left overnight to adhere. PBMCs or granulocytes (7.5*10^5^ cells/well), isolated just before the experiment, were seeded in 96-well U-bottom plates. All cells were incubated in the corresponding fresh medium containing different concentrations of peptides. After 24 hours the medium was replaced by 100 μl culture medium containing 10% WST-1 (Roche, Basel, Switzerland). Colorimetric changes were measured, after 20 (RAW264.7 cells) or 60 min (3D4/31 cells, PBMCs, and granulocytes) incubation at 450 nm using a FLUOstar Omega microplate reader (BMG Labtech GmbH, Ortenberg, Germany). Cell viability was calculated as percentage viability with the no peptide control set to 100% viability.

### Hemolysis

RBCs (2.25*10^7^ cells/well) were tested in V-bottom plates and mixed with different peptides concentrations (0.63–40 μM) and incubated for 60 min at 37 °C. Plates were centrifuged 2 min at 1360xg (with slow brake) and 75 μl supernatant was transferred to a flat bottom plate to measure absorbance at 405 nm using a FLUOstar Omega microplate reader. The percent of hemolysis was calculated using a negative (no peptide) and a positive (0.2% Triton X-100) control, 0% and 100% lysis, respectively.

### LPS stimulation

RAW264.7 cells (5*10^4^ cells/well) were seeded in a 96-wells tissue culture treated microtiter plate and left overnight to adhere. The cells were incubated for 24 h in fresh medium containing different concentration peptides and 100 ng/ml UltraPure LPS *E*. *coli* O111:B4 (TLR-4) (InVivoGen, San Diego, CA, USA). The supernatant was collected and stored at −20 °C until further use. TNFα release was used as an indication for cell activation and measured using a Duoset ELISA kit (R&D systems, Minneapolis, MN, USA). ELISAs were performed according manufacturer’s instructions.

Nitric oxide (NO) production was measured with the Griess assay. Supernatants (50 µl) were incubated with 50 µl 1% sulfanilamide in 85% phosphoric acid for 5 min at RT in the dark. N-(1-Naphthyl)ethylenediamine dihydrochloride (NED; 0.1%) (50 µl) was added to the mixture and incubated for another 5 min, after which the colorimetric changes were measured at 550 nm using a FLUOstar Omega microplate reader. Concentration NO was calculated using a standard curve.

### Isothermal titration calorimetry

Isothermal titration calorimetry (ITC) was performed with the Low Volume NanoITC (TA Instruments-Waters LLC, New Castle, DE, USA) to determine interaction between LPS and the different peptides. 100 μM UltraPure LPS *E*. *coli* O111:B4 was diluted in MilliQ, after which 4x dilution in dPBS (Gibco) was made. Peptides were diluted to 800 μM in MilliQ, followed by 4-fold dilution in dPBS. The chamber was filled with 164 μl LPS, the peptide was titrated into the chamber with 1.96 μl every 300 seconds. Experiments were performed at 37 °C and analyzed using the Nano Analyze software (TA instruments-Waters LLC). All experiments were performed 2 times and corrected for buffer in buffer titration, after which the both experiments were averaged. Two independent models were used, except for LL-37 with one independent model fit, to determine the peptide-LPS interaction.

### Tris-Tricine SDS-PAGE

P7 or mP7 (1 μg) was prepared in sample buffer (25 mM Tris-HCL, 2% SDS (w/v), 0.01% (w/v) bromophenol blue) and heated (5 min, 95 °C). If indicated, samples were reduced by addition of 25 mM DTT to the sample buffer. Peptides were separated on an SDS-PAGE gel (Running gel; 18% (Bis)acrylamide (BioRad), 1 M Tris-HCL, 0.1% SDS (w/v), 10% glycerol (v/v), 0.1% ammonium persulfate (APS) (w/v), 0.04% TEMED (v/v) (BioRad) and Stacking gel; 4% (Bis)acrylamide, 1 M Tris-HCL, 0.1% SDS (w/v), 0.1% APS (w/v), 0.04% TEMED (v/v)). Directly after running (20 min, 50 V) followed by 120 min, 50 mA) gels were fixed in 5% glutaraldehyde for 60 minutes. After extensive washing with MilliQ, proteins were stained-in-gel with Coomassie blue solution (50% (v/v) methanol, 7% acetic acid, 0.1% (w/v) Coomassie blue). Background staining was removed with multiple washing steps with destaining solution (50% (v/v) methanol, 7% acetic acid) until peptide bands were properly visible. Images were made using ChemiDoc^TM^ MP Universal hood III (BioRad) and analyzed with the corresponding ImageLab^TM^ software (version 5.2.1.). Protein weight was calculated with: http://www.sciencegateway.org/tools/proteinmw.htm, presented in Table 1.

## Results

### PMAP-36, CATH-2 and LL-37 efficiently kill *E*. *coli* O78 via different mechanisms

**Figure 1:**
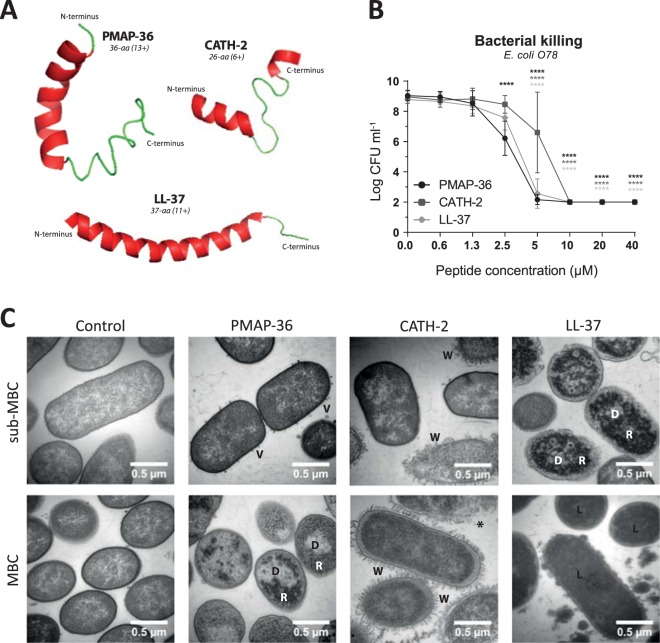
PMAP-36, CATH-2 and LL-37 efficiently kill *E*. *coli* O78 but in a different way. Structures of PMAP-36, CATH-2 and LL-37 as predicted using I-TASSER^46–48^ **(A)**. Bacterial killing of 10^6^ CFU ml^−1^ *E*. *coli* O78 by PMAP-36, CATH-2 and LL-37 was tested using a colony count assay **(B)**. Peptide dependent morphological changes of *E*. *coli* O78 were determined by TEM. Representative images are shown for the MBC value and 4x below MBC value (sub-MBC). V – vesicle; D – clustered DNA; R – clustered ribosomes; W – detached and wrinkled membrane; * - ruptured cell; L – lysed cell **(C)**. Data is plotted as average +/− s.d. (N = 3–4). Samples were compared to the no peptide control, using two-way ANOVA with the Bonferroni post-hoc test. (*p ≤ 0.05; **p ≤ 0.01; ***p ≤ 0.001; ****p ≤ 0.0001; black - PMAP-36; dark gray – CATH-2; light gray – LL-37).

Throughout this study we used two well-studied cathelicidins, human LL-37 and chicken CATH-2, to compare the properties of PMAP-36. LL-37 adopts to an α-helix of almost the entire peptide, while CATH-2 is characterized by two short α-helical fragments that are connected by a proline-induced hinge region. PMAP-36 forms an α-helix on the N-terminal side which is broken by a proline-induced hinge region. The C-terminal part seems more loosely structured (Fig. 1A). LL-37 (6+) and PMAP-36 (13+) are 37 and 36 amino acids long, whereas CATH-2 (8+) is slightly shorter with 26 amino acids (Table 1).

PMAP-36, CATH-2 and LL-37 had strong antibacterial activity against *E*. *coli* O78, with an MBC between 5 and 10 μM. PMAP-36 was the most active peptide, with a significant inhibition at a concentration of 2.5 μM (Fig. 1B). Morphological changes of *E*. coli O78 were determined using transmission electron microscopy (TEM). MBC values were determined for the higher concentration bacteria required for TEM experiments, to be able to analyze the peptide effect at MBC and sub-MBC (*i*.*e*. ¼ MBC). Control cells contained intact membranes and showed an even distribution of DNA and ribosomes, *i*.*e*. the light and darker areas respectively. PMAP-36 caused release of small vesicles at sub-MBC (5 μM). At MBC concentration (20 μM) no vesicle release was observed anymore; however, DNA and ribosomes were clustered and located close to the inner membrane of the bacteria. CATH-2 affected several, but not all, bacteria at sub-MBC (10 μM) and all bacteria at MBC (40 μM). CATH-2 caused membrane wrinkling or even complete membrane rupture. Sub-MBC of LL-37 (40 μM) caused clustering of the DNA and ribosomes. At MBC (160 μM), LL-37 lysed the bacteria, causing all the staining to enter the bacteria, leading to loss of contrast.

### PMAP-36, CATH-2 and LL-37 are mildly cytotoxic to mammalian cells

**Figure 2:**
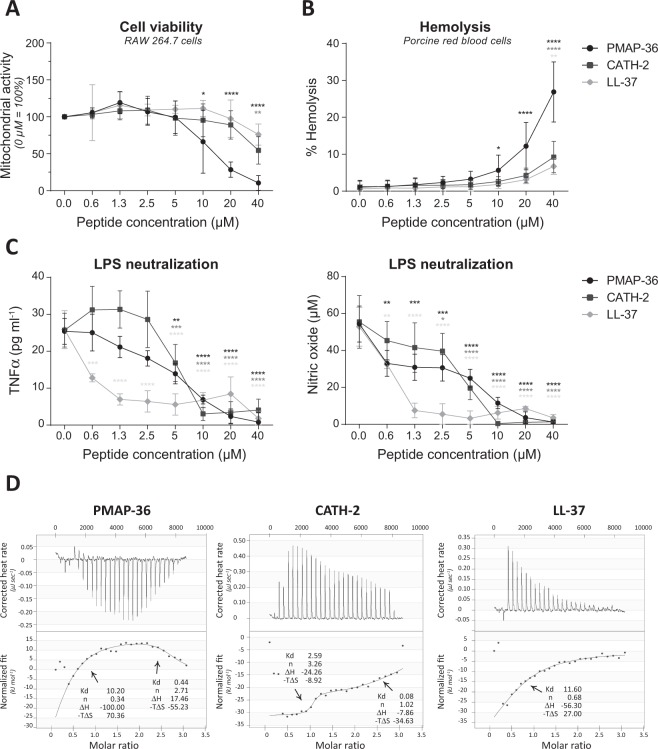
PMAP-36, CATH-2 and LL-37 exhibit different LPS binding characteristics. Cytotoxic effects against RAW264.7 cells were tested using a WST-1 assay, indicating cell viability. No peptide control was set to 100% cell viability **(A)**. Hemolytic effects on porcine red blood cells were tested by determining heme release. Triton (0.2% v/v) was set to 100% lysis and no peptide as negative control **(B)**. Peptides were mixed with 100 ng ml^−1^ LPS O111:B4 for 5–10 minutes, before cells were stimulated with this mixture. TNFα and NO production were used to measure cell activity **(C)**. Thermodynamic binding capacity of the peptides was measured with isothermal titration calorimetry (ITC). Every 300 seconds, 1.96 μl peptide solution (200 μM) was titrated into 164 μl LPS solution (25 mM). Heat evolved was measured (top panel) and normalized integrated heat was plotted against the molar ratio between LPS and the peptides (lower panel). Two independent models were used to fit the data and to calculate K_d_ (μM), the amount of peptide that binds to LPS (n), ΔH (kJ mol^−1^) and −TΔS (kJ mol^−1^). Experiments (N = 2) were corrected for heat change of dilution (buffer into buffer titration) and averaged before plotting and model fitting (**D**). Data is plotted as average +/− s.d. with N = 5–7 (**A**,**B**) or N = 3 (**C**). Samples were compared to the no peptide control, using two-way ANOVA with the Bonferroni post-hoc test. (*p ≤ 0.05; **p ≤ 0.01; ***p ≤ 0.001; ****p ≤ 0.0001; black - PMAP-36; dark gray – CATH-2; light gray – LL-37).

LL-37 is produced by many cell types, like macrophages, NK-cells, neutrophils, and epithelial cells^27^, whereas in chickens CATH-2 expression is highly specific for heterophils (chickens granulocytes resembling mammalian neutrophils)^41^. Expression of PMAP-36 was found in neutrophil secretions^37^, although it might be expressed in other cell types as well. Because all three peptides are positively charged and amphipathic they may also interact with mammalian cell membranes. Therefore, the cytotoxic effect of the peptides was tested on several types of mammalian cells. LL-37 and CATH-2 proved to be mildly cytotoxic to murine RAW264.7 macrophage-like cells, whereas PMAP-36 was quite harmful to these cells, although at higher concentrations than needed for killing *E*. *coli* (Fig. 2A). Only the highest concentration of PMAP-36 (40 µM) was slightly cytotoxic for porcine 3D4/31 macrophage-like cells (Fig. S1A). Due to the fact that these cells were unresponsive to stimulation to induce cytokine expression (data not shown), this cell line was not used for further experiments. PMAP-36 caused up to 30% hemolysis of porcine erythrocytes, whereas CATH-2 and LL-37 caused less than 10% of the erythrocytes to lyse (Fig. 2B). Porcine granulocytes were resistant to any cytotoxic effect of the peptides, whereas porcine PBMCs were slightly affected by 20–40 μM PMAP-36, but not by CATH-2 and LL-37 (Fig. S1B).

### PMAP-36, CATH-2 and LL-37 exhibit different LPS binding characteristics

LL-37 was described to inhibit LPS-induced cytokine production^3,42,43^, thereby protecting against endotoxin shock *in vivo*^15^, similar to CATH-2^3,29,34^, and PMAP-36^3^. In this study, the capability to inhibit LPS-induced TNFα secretion by RAW264.7 cells was compared in a standardized assay. LL-37 was most efficient in inhibiting LPS-induced macrophage activation, causing a significant reduction of LPS-activation at the lowest concentration tested (0.63 μM). At higher concentrations, CATH-2 and PMAP-36 were also able to completely block TNFα secretion at 10 μM (Fig. 2C). In addition to TNFα, cells secrete nitric oxide (NO) in response to LPS. NO can directly react with superoxide, producing peroxynitrite, a powerful oxidant with potent antimicrobial activity; yet, at high levels NO may cause tissue damage and cell death^44^. NO production in response to LPS was inhibited by all three peptides. At low concentrations, CATH-2 and PMAP-36 inhibited the LPS-induced NO production more efficiently than LPS-induced TNFα secretion (Fig. 2C).

The LPS binding capacity of peptides was tested using isothermal titration calorimetry (ITC) to study whether the inhibition of LPS-induced activation can be (partially) explained by direct LPS binding and neutralization^45^. Although all three peptides were very effective in inhibiting LPS-activation, they bind in a very different manner to LPS (Fig. 2D). PMAP-36 and CATH-2 bind in a biphasic manner, whereas LL-37 binds in a monophasic way. PMAP-36 binds in an enthalpy- or entropy-driven manner, depending on the binding phase. CATH-2 binds in an enthalpy- and entropy-manner in both phases. LL-37 binds only in an enthalpy-driven manner. The three peptides differ also in the stoichiometry and dissociation coefficient (K_d_). LL-37 has a high K_d_, indicating weak LPS binding. Each LL-37 molecule binds less than one LPS molecule. Both PMAP-36 and CATH-2 bind LPS stronger in the second binding phase, indicated by the lower K_d_. The binding ratio of PMAP-36 to LPS is close to 1:3, whereas that of CATH-2 to LPS is 1:1. It should be noted, that PMAP-36 forms dimers by S-S bridging of the cysteine on the C-terminal side of the peptide. However, in this paper, the molecular weight of PMAP-36 is calculated as monomer. Therefore, it can be stated that one PMAP-36 dimer molecule ((PMAP-36)_2_) binds to 1.35 LPS molecules.

### Antibacterial and immunomodulatory effects of PMAP-36 are not affected by deletion of the first 11 N-terminal amino acids

**Figure 3:**
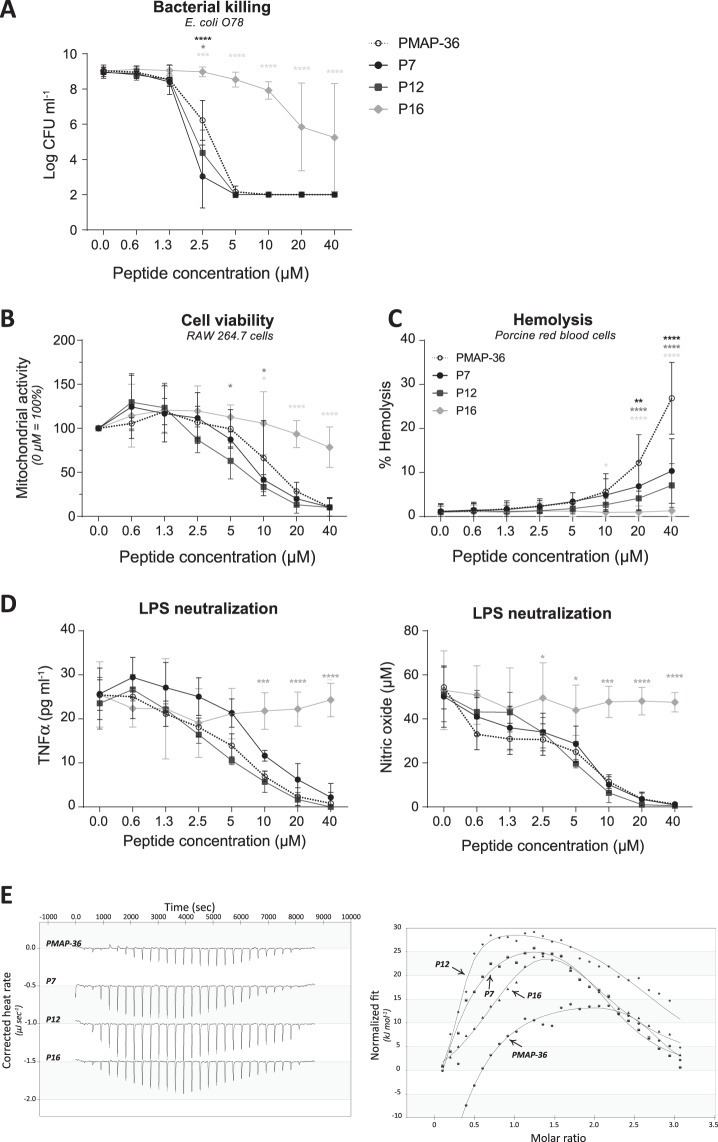
Antibacterial and immunomodulatory effects of PMAP-36 are not affected by deletion of the first 11 N-terminal amino acids. Bacterial killing of PMAP-36 and analogs with N-terminal deletions were tested for their *E*. *coli* O78 killing capacities **(A)**. Cytotoxic effects of the peptide analogs to RAW264.7 cells were determined using WST-1 reagent, indicating cell viability. No peptide control was set to 100% cell viability **(B)**. Hemolytic effects on porcine red blood cells were determined by heme release. The positive control, 0.2% Triton was set to 100% lysis and no peptide control as background lysis **(C)**. The PMAP-36 analogs were tested for their ability to neutralize LPS O111:B4. TNFα and NO production were used as measures for cell activation **(D)**. Thermodynamic binding of peptides and LPS O111:B4 was measured with isothermal calorimetry (ITC). Every 300 seconds, 1.96 μl peptide solution (200 μM) was titrated into 164 μl LPS solution (25 mM). Heat evolved was measured (left panel) and normalized integrated heat was plotted against the molar ratio between LPS and the peptides (right panel). Two independent models were used to fit the data and calculate K_d_ (μM), the amount of peptide that binds to LPS (n), ΔH (kJ mol^−1^) and −TΔS (kJ mol^−1^). Experiments (N = 2) were corrected for heat change of dilution (buffer into buffer titration) and averaged before plotted and model fitting **(E)**. Data is plotted as average +/− s.d. with N = 3–7 **(A)**, N = 5–7 **(B**,**C)** and N = 3 **(D)**. Samples were compared to PMAP-36 full-length peptide in the same concentration, using two-way ANOVA with the Bonferroni post-hoc test. (*p ≤ 0.05; **p ≤ 0.01; ***p ≤ 0.001;****p ≤ 0.0001; black – P7; dark gray – P12; light gray – P16).

N-terminally truncated PMAP-36 derivatives were produced in order to potentially obtain PMAP-36 based peptides with lower toxicity but retaining antibacterial and /or immunomodulatory activity. Peptides were made with N-terminal truncations of either 6, 11 or 15 amino acids (peptides P7, P12 and P16), thereby reducing the long cationic α-helical N-terminal tail of the peptide and the net charge of the peptides (*e*.*g*. 10+, 6+, 3+ respectively). According to I-TASSER structure prediction^46–48^, the truncated peptides are still able to form an α-helix at the N-terminus. Analysis of the antimicrobial activity of the shorter derivatives showed that the first 11 amino acids were not essential for killing of *E*. *coli* O78. P7 and P12 were even slightly more efficient than the full-length peptide. However, a truncation with 15 N-terminal amino acids strongly reduced the efficacy of killing *E*. *coli* O78 resulting in a MBC > 40 μM (Fig. 3A). Similar to the antibacterial activity, cytotoxicity against RAW264.7 cells was not changed by truncation with 6 or 11 N-terminal amino acids, whereas depletion of 15 amino acids strongly reduced the cytotoxicity (Fig. 3B). Interestingly, truncation with 6 N-terminal amino acids reduced hemolysis to only 10% (similar to CATH-2 and LL-37) at the highest peptide concentration. Deletion of more N-terminal amino acids even reduced hemolysis further, with a complete lack of hemolysis for P16 (Fig. 3C).

Finally, deletion of the first 6 or 11 N-terminal amino acids did not affect the LPS neutralization capacity of PMAP-36. Both TNFα secretion and NO production of RAW264.7 cells were as strongly inhibited by P7 and P12 as was observed for the full-length peptide. However, P16 completely lost its LPS neutralizing capacity (Fig. 3D). Interestingly, although unable to neutralize LPS activation, P16 still bound strongly to LPS as shown with ITC. The three analogs showed a similar LPS binding mechanism as the full-length peptide. All peptides showed biphasic binding with an initial enthalpy-driven binding and a second more entropy-driven binding event, although there were some relatively small differences in binding parameters (Fig. 3E and Table 2).

**Table 2:** Thermodynamics of peptides binding to LPS O111:B4.

Peptide	*First binding event*				*Second binding event*			
	K_d_ (μM)	n	ΔH	−TΔS	K_d_ (μM)	n	ΔH	−TΔS
PMAP-36*	10.20	0.337	−100.00	70.36	0.44	2.706	17.46	−55.23
P7*	12.27	0.305	−97.69	68.53	1.60	2.168	35.31	−69.72
P12*	1.46	0.300	−38.60	3.95	2.68	2.763	32.73	−65.81
P16*	5.12	1.069	−67.54	34.17	4.03	1.979	62.37	−94.4
mP1	0.57	0.509	−27.06	−10.03	1.48	1.650	27.81	−62.42
mP7	0.24	0.392	−26.23	−13.09	0.34	1.499	27.37	−66.12
mP12	2.43	0.347	−100.00	66.66	4.29	1.214	84.57	−116.50
mP16	1.91	0.371	−91.09	57.13	6.44	0.751	99.95	−130.80

Overview of ITC results of the binding capacity of (analogs of) PMAP-36 to LPS O111:B4. K_d_ – dissociation coefficient (μM); n – number of peptide molecules binding to one LPS molecule; ΔH – enthalpy changes (kJ/mol); −TΔS – entropy changes (J/mol·K).

*Note: PMAP-36, P7, P12, and P16 are calculated as monomers, although they form dimers in solution.

### PMAP-36 monomeric analogs have similar antimicrobial activities, but have reduced LPS neutralizing capacities

**Figure 4:**
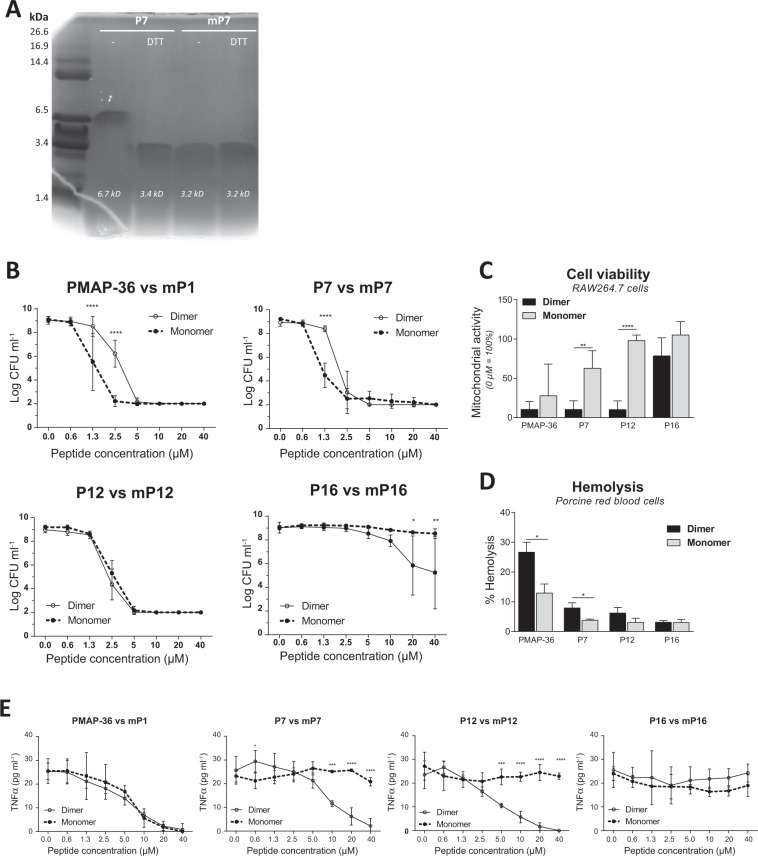
PMAP-36 monomeric analogs have similar antimicrobial activities, but have reduced LPS neutralizing capacities. Reduced (DTT) or non-reduced P7 and mP7 were separated with an 18% Tris-Tricine SDS-PAGE gel and stained with Coomassie blue solution. The gel image is not cropped. **(A)**. Differences between monomeric and dimeric PMAP-36 analog equivalents were tested for *E*. *coli* O78 killing **(B)**. Cytotoxic effects of 40 μM dimeric or monomeric PMAP-36 analogs on RAW264.7 cells **(C)**, or porcine red blood cells **(D)**. Capability of PMAP-36 analogs to neutralize 100 ng ml^−1^ LPS O111:B4 **(E)**. Data is plotted as average +/− s.d. with N = 3–7 **(B**–**D)**. Results of monomeric peptides were compared to its dimeric equivalent peptide in the same concentration, using two-way ANOVA with the Bonferroni post-hoc test. (*p ≤ 0.05; **p ≤ 0.01; ***p ≤ 0.001; ****p ≤ 0.0001).

As mentioned before, PMAP-36 contains a cysteine at position 35 by which it can form homodimers by disulfide bonding. Structural changes caused by dimerization may affect the activity of the peptide. Therefore, the effects of dimerization were studied in more depth. The cysteine at position 35 was replaced by a serine and the last amino acid was deleted (Table 1). This efficiently prevented the dimerization of the PMAP-36 analogs (Fig. 4A). The molecular weight, and therefore the molarity, of PMAP-36, was calculated on a monomer basis, *i*.*e*. 1 μM (PMAP-36)_2_ is depicted as 2 μM of PMAP-36 subunits in the figures.

It was found that the antibacterial activity against *E*. *coli* of the monomeric analogs of PMAP-36 and P7 (mP1 and mP7 respectively) was slightly increased compared to their dimeric counterparts (Fig. 4B**)**. The antibacterial activity of mP12 was identical to P12, whereas mP16 lost its antibacterial activity completely (Fig. 4B). Prevention of the dimerization greatly reduced the cytotoxicity against RAW264.7 cells, especially for mP7 and mP12, which hardly show cytotoxicity at all (Figs 4C and S2A). Similar effects were observed for the hemolytic activity of monomers. Only mP1 had some hemolytic effect at 40 μM, whereas the other analogs were not hemolytic at all (Figs 4D and S2B). Although mP1 was less cytotoxic and less hemolytic compared to PMAP-36, the monomer was as efficient as the full-length peptide with respect to LPS neutralization. mP7 and mP12 exhibited only low cytotoxicity but also strongly reduced LPS neutralizing capacity (Figs 4E and S2C). However, this was not due to a lack of binding to LPS, since ITC results showed an even stronger binding to LPS for all monomeric analogs. However, in the second binding event the binding was slightly less strong compared to the dimeric analogs (Table 2).

## Discussion

The prevalence of antibiotic resistance against conventional antibiotics is ever increasing and we are about to enter the post-antibiotic era. In many countries the use of low doses of antibiotics to promote growth of livestock has been banned. However, despite these efforts huge amounts of antibiotics are still utilized to prevent outbreaks of disease in farm animals, in particular in pigs and poultry^49^. To effectively reduce the use of antibiotics in veterinary medicine, alternative strategies and drugs are needed to prevent and treat diseased animals. In this study the antibacterial and immunomodulatory activities and mechanisms of porcine cathelicidin PMAP-36 were examined and compared to those of human cathelicidin LL-37 and chicken cathelicidin-2. Additionally, the effect of PMAP-36 dimerization and/or truncation was examined.

In the pig, 11 different cathelicidins are found^4^, of which PMAP-36 is phylogenetically closest to LL-37^21^ and may therefore fulfill a similar versatile role in the porcine innate immune system. Our study revealed that PMAP-36, LL-37 and CATH-2 were equally efficient in *E*. *coli* killing exhibiting similar MBCs, although by different modes of actions, as shown by TEM. At sub-MBC of LL-37 clustering of DNA and ribosome was observed, whereas at MBC all cells are lysed. This corresponds with the idea that LL-37 first attacks the outer membrane of *E*. *coli* leading to toroidal pore formation^50^, and eventually translocation to the periplasmic space^51^. CATH-2 on the other hand, attacks the bacterial membrane already at sub-MBC. Most likely CATH-2 binding to the membrane itself catalyzes the binding of other CATH-2 molecules, since not all bacteria are affected at sub-MBC. Previously, it was shown that CATH-2 may translocate intracellularly at sub-MBC levels^6^, indicating that membrane disruption at sub-MBC allows the peptide to enter the cell. In contrast to LL-37 and CATH-2, sub-MBC concentrations of PMAP-36 causes vesicle release by *E*. *coli*. At MBC PMAP-36 causes, similar to LL-37, cell lysis and clustering of DNA and ribosomes. PMAP-36 and LL-37 have been previously shown to disrupts the inner membrane, while CATH-2 causes far less inner membrane disruption yet is able to translocate intracellularly at sub-MBC levels^3,6^. Vesicle formation by Gram-negative bacteria is considered a survival strategy to cope with host-associated stressors and may be the result of compromised membrane lipid asymmetry, accumulation of misfolded proteins in the outer membrane or induced LPS modifications^52^.

Since LPS is a main component of the Gram-negative bacterial cell wall, the effects of the peptides on LPS-mediated activation and binding to LPS were investigated. All three peptides inhibited LPS-induced macrophage activation, although LL-37 was most effective reducing macrophage activation for 50% at only 0.63 µM, whereas 5 µM of CATH-2 or PMAP-36 was needed for this reduction. Interestingly, ITC analysis showed that LL-37 binds LPS only weakly in contrast to CATH-2 and PMAP-36 that showed a strong LPS binding capacity, suggesting a different mechanism for inhibition of LPS-induced macrophage activation. LL-37 is shown to enhance LPS uptake by endocytosis and directs the LPS towards the lysosomes for degradation without cell activation in liver endothelial cells^53^. Another study has shown that LL-37 inhibits the LPS activation by preventing the formation of the CD14-TLR-4 complex and thereby prevent cell activation^28^. However, whereas LL-37 suppresses the inflammatory responses, it did not block genes required for cell migration. It might suppress the translocation of NF-kB to the nucleus leading to a more general anti-inflammatory effect^54^. Indeed, LL-37 was also shown to inhibit the activation of macrophages by lipoteichoic acid (LTA) (TLR-2), although activation with DNA (TLR-9) was not inhibited^3^.

LL-37 binds via hydrogen bonding, similar to CATH-2 although CATH-2 also shows hydrophobic binding at higher concentrations. Hydrophobic interaction between peptide and membrane is often correlated with membrane disruption^55^. CATH-2 was previously shown to interact with smooth LPS, but not with rough LPS. Lipid A can also be bound by CATH-2^6^. This suggests that CATH-2 binds LPS initially via the O-antigen and at higher concentrations disrupt the LPS micelles and binds lipid A by hydrophobic binding. In the case of rough LPS, which lacks the O-antigen, preventing the initial CATH-2 binding by which CATH-2 cannot disrupt the LPS micelles and therefore CATH-2 is unable to interact with rough LPS. The binding mechanism of PMAP-36 to LPS is more complex. Initially, PMAP-36 binding depends solely on hydrogen bonding and shifts towards hydrophobic interactions when PMAP-36 accumulates. It should be taken into account that PMAP-36 binding to LPS is complex, and it cannot be determined from our data whether the 2 binding events are happening simultaneously or subsequently which would affect binding parameters in our modelling. Additional techniques should be performed to unravel the exact binding mechanism of PMAP-36.

To study the function of PMAP-36 in more detail, several analogs were made. PMAP-36 contains 13 cationic residues, mainly at the N-terminal part of the peptide, and several hydrophobic residues, mainly at the C-terminal end. Previously it was shown that deletion of 12 N-terminal or C-terminal residues had no to little effect on the direct antimicrobial activity of the peptides^38,39^; however, the immunomodulatory capacity was not studied. In the current study, the N-terminal end of PMAP-36 was systematically truncated. N-terminal truncation of 11 residues, and thereby reduction in size (25aa) and charge (6+) had only a minor impact on the efficiency of *E*. *coli* killing. Deletion of an additional 4 residues, resulting in a charge of 3+, strongly reduced the antibacterial capacity. One explanation is that PMAP-36 needs to span the membrane in order to kill *E*. *coli*, for which a peptide needs approximately 15–20 amino acids, depending on the structure of the peptide and thickness of the membrane^56^. Truncation of the first 15 residues leaves a PMAP-36 analog of only 21 amino acids, which would theoretically just be able to span the membrane. As shown previously by Coorens *et al*. PMAP-36 permeabilizes the membrane of *E*.*coli* upon killing^3^. The N-terminus of the truncated peptides are predicited by I-TASSER^46–48^ to adopt to an α-helix, just like the full length peptide. Formation of the α-helix might prevent the shortest peptide to fully span the membrane. Therefore, it would be interesting to study the inner membrane permeabilization for the PMAP-36 truncated peptides. Another explanation might be the charge reduction, since the peptide might lose its antibacterial activity if the net charge drops below 5+^57^.

PMAP-36 contains a cysteine residue on position 35, causing the peptide to dimerize by disulfide bonding. Previously, Scocchi *et al*.^36^. tested the antibacterial activity of monomeric versus dimeric PMAP-36 and observed only minor effects, comparable to our results. However, in our study, also monomers of shorter PMAP-36 analogs were studied. Similar to the dimer, N-terminal truncation of 11 residues in the PMAP-36 monomer had only a minor effect on *E*. *coli* killing. Additional truncation of 4 N-terminal residues completely blocked the antibacterial capacity.

Besides antibacterial activity, for the first time the immunomodulatory aspects of PMAP-36 and its analogs were studied. Similar to its antimicrobial activity, deletion of 11 N-terminal residues had only minor effects on the cytotoxicity and LPS neutralization, whereas these effects were strongly inhibited by deletion of an additional 4 N-terminal residues. This reduction in cytotoxicity is most likely due to the reduction in charge, as shown by Dathe *et al*.^57^. However, if only 6 N-terminal residues were depleted of the monomeric version of PMAP-36, neither cytotoxicity nor LPS neutralization capacity was observed, indicating that PMAP-36 needs to dimerize in order to display its full immunomodulatory capacity. In contrast, all PMAP-36 analogs, including the inactive P16 and mP16 peptides, were found to have strong LPS binding capacities. These results suggest that PMAP-36 binds LPS and thereby shields it from recognition by TLR-4. Short and monomeric PMAP-36 analogs are unable to prevent LPS-induced activation of macrophages, despite their strong binding to LPS. Whether PMAP-36 inhibits LPS activation in a direct manner by shielding LPS from TLR-4 recognition or in an indirect manner for which it needs a certain charge, length or a propensity to form dimers, an explanation that cannot be excluded based on this data.

In this study we have compared the porcine cathelicidin PMAP-36 to two well-studied cathelicidins, human LL-37 and chicken CATH-2. Although the three peptides kill *E*. *coli* at similar concentrations, they have a different mode of action. Further, the function of PMAP-36 was studied in more detail by designing several analogs. This study showed that the first 11 N-terminal residues are dispensable for both the antibacterial as the immunomodulatory functions of PMAP-36. On the other hand, if dimerization of the peptide is prevented by deletion of the cysteine at the C-terminal site of the peptide, the immunomodulatory activity of the peptide is lower, especially in combination with N-truncations. Therefore, it can be concluded that dimerization is especially needed for the immunomodulatory activity of the truncated peptides, in contrast to the requirement of the first N-terminal residues. The specific properties of PMAP-36 and its analogs may serve as paradigms to develop alternatives to antibiotics.

## Supplementary information

Supplementary figures

## References

[1] Zasloff M (2002). Antimicrobial peptides in health and disease. N Engl J Med.

[2] Vandamme D, Landuyt B, Luyten W, Schoofs L (2012). A comprehensive summary of LL-37, the factotum human cathelicidin peptide. Cell Immunol.

[3] Coorens M, Scheenstra MR, Veldhuizen EJ, Haagsman HP (2017). Interspecies cathelicidin comparison reveals divergence in antimicrobial activity, TLR modulation, chemokine induction and regulation of phagocytosis. Sci Rep.

[4] Zhang G, Ross CR, Blecha F (2000). Porcine antimicrobial peptides: new prospects for ancient molecules of host defense. Vet Res.

[5] Coorens M, van Dijk A, Bikker F, Veldhuizen EJ, Haagsman HP (2015). Importance of Endosomal Cathelicidin Degradation To Enhance DNA-Induced Chicken Macrophage Activation. J Immunol.

[6] Schneider VA (2016). Imaging the antimicrobial mechanism(s) of cathelicidin-2. Sci Rep.

[7] Veldhuizen, E. J. A. *et al*. Antimicrobial and immunomodulatory activity of PMAP-23 derived peptides. *Protein Pept Lett*, 10.2174/0929866524666170428150925 (2017). 10.2174/0929866524666170428150925 28462713

[8] Currie SM (2013). The human cathelicidin LL-37 has antiviral activity against respiratory syncytial virus. PLoS One.

[9] Harcourt JL (2016). Human cathelicidin, LL-37, inhibits respiratory syncytial virus infection in polarized airway epithelial cells. BMC Res Notes.

[10] Tripathi S (2013). The human cathelicidin LL-37 inhibits influenza A viruses through a mechanism distinct from that of surfactant protein D or defensins. J Gen Virol.

[11] Benincasa M (2006). Fungicidal activity of five cathelicidin peptides against clinically isolated yeasts. J Antimicrob Chemother.

[12] Cauchard S (2016). Killing of Trypanozoon Parasites by the Equine Cathelicidin eCATH1. Antimicrob Agents Chemother.

[13] Rico-Mata R, De Leon-Rodriguez LM, Avila EE (2013). Effect of antimicrobial peptides derived from human cathelicidin LL-37 on Entamoeba histolytica trophozoites. Exp Parasitol.

[14] De Y (2000). LL-37, the neutrophil granule- and epithelial cell-derived cathelicidin, utilizes formyl peptide receptor-like 1 (FPRL1) as a receptor to chemoattract human peripheral blood neutrophils, monocytes, and T cells. J Exp Med.

[15] Scott MG, Davidson DJ, Gold MR, Bowdish D, Hancock RE (2002). The human antimicrobial peptide LL-37 is a multifunctional modulator of innate immune responses. J Immunol.

[16] Lishko VK, Moreno B, Podolnikova NP, Ugarova TP (2016). Identification of Human Cathelicidin Peptide LL-37 as a Ligand for Macrophage Integrin alphaMbeta2 (Mac-1, CD11b/CD18) that Promotes Phagocytosis by Opsonizing Bacteria. Res Rep Biochem.

[17] Wan M (2014). Antimicrobial peptide LL-37 promotes bacterial phagocytosis by human macrophages. J Leukoc Biol.

[18] Zhang X, Bajic G, Andersen GR, Christiansen SH, Vorup-Jensen T (2016). The cationic peptide LL-37 binds Mac-1 (CD11b/CD18) with a low dissociation rate and promotes phagocytosis. Biochim Biophys Acta.

[19] Molhoek EM, van Dijk A, Veldhuizen EJ, Haagsman HP, Bikker FJ (2011). Improved proteolytic stability of chicken cathelicidin-2 derived peptides by D-amino acid substitutions and cyclization. Peptides.

[20] Okuda D, Yomogida S, Tamura H, Nagaoka I (2006). Determination of the antibacterial and lipopolysaccharide-neutralizing regions of guinea pig neutrophil cathelicidin peptide CAP11. Antimicrob Agents Chemother.

[21] Baumann A, Demoulins T, Python S, Summerfield A (2014). Porcine cathelicidins efficiently complex and deliver nucleic acids to plasmacytoid dendritic cells and can thereby mediate bacteria-induced IFN-alpha responses. J Immunol.

[22] Lande R (2007). Plasmacytoid dendritic cells sense self-DNA coupled with antimicrobial peptide. Nature.

[23] van der Does AM (2010). LL-37 directs macrophage differentiation toward macrophages with a proinflammatory signature. J Immunol.

[24] Cuperus T, Coorens M, van Dijk A, Haagsman HP (2013). Avian host defense peptides. Developmental and Comparative Immunology.

[25] Mishra B, Epand RF, Epand RM, Wang G (2013). Structural location determines functional roles of the basic amino acids of KR-12, the smallest antimicrobial peptide from human cathelicidin LL-37. RSC Adv.

[26] Fabisiak A, Murawska N, Fichna J (2016). LL-37: Cathelicidin-related antimicrobial peptide with pleiotropic activity. Pharmacol Rep.

[27] Kahlenberg JM, Kaplan MJ (2013). Little peptide, big effects: the role of LL-37 in inflammation and autoimmune disease. J Immunol.

[28] Verjans ET, Zels S, Luyten W, Landuyt B, Schoofs L (2016). Molecular mechanisms of LL-37-induced receptor activation: An overview. Peptides.

[29] van Dijk A (2009). Identification of chicken cathelicidin-2 core elements involved in antibacterial and immunomodulatory activities. Mol Immunol.

[30] Molhoek EM (2010). Chicken cathelicidin-2-derived peptides with enhanced immunomodulatory and antibacterial activities against biological warfare agents. Int J Antimicrob Agents.

[31] Veldhuizen EJ, Brouwer EC, Schneider VA, Fluit AC (2013). Chicken cathelicidins display antimicrobial activity against multiresistant bacteria without inducing strong resistance. PLoS One.

[32] van Dijk A (2009). Chicken heterophils are recruited to the site of Salmonella infection and release antibacterial mature Cathelicidin-2 upon stimulation with LPS. Mol Immunol.

[33] Ordonez SR, Amarullah IH, Wubbolts RW, Veldhuizen EJ, Haagsman HP (2014). Fungicidal mechanisms of cathelicidins LL-37 and CATH-2 revealed by live-cell imaging. Antimicrob Agents Chemother.

[34] van Dijk A (2016). Immunomodulatory and Anti-Inflammatory Activities of Chicken Cathelicidin-2 Derived Peptides. PLoS One.

[35] Storici P, Scocchi M, Tossi A, Gennaro R, Zanetti M (1994). Chemical synthesis and biological activity of a novel antibacterial peptide deduced from a pig myeloid cDNA. FEBS Lett.

[36] Scocchi M (2005). Structural aspects and biological properties of the cathelicidin PMAP-36. FEBS J.

[37] Scapinello S (2011). Bactericidal activity of porcine neutrophil secretions. Vet Immunol Immunopathol.

[38] Lv Y (2014). Antimicrobial properties and membrane-active mechanism of a potential alpha-helical antimicrobial derived from cathelicidin PMAP-36. PLoS One.

[39] Lyu Y, Yang Y, Lyu X, Dong N, Shan A (2016). Antimicrobial activity, improved cell selectivity and mode of action of short PMAP-36-derived peptides against bacteria and Candida. Sci Rep.

[40] van Dijk A (2007). The beta-defensin gallinacin-6 is expressed in the chicken digestive tract and has antimicrobial activity against food-borne pathogens. Antimicrob Agents Chemother.

[41] Cuperus T, van Dijk A, Dwars RM, Haagsman HP (2016). Localization and developmental expression of two chicken host defense peptides: cathelicidin-2 and avian beta-defensin 9. Dev Comp Immunol.

[42] Bowdish DM, Davidson DJ, Scott MG, Hancock RE (2005). Immunomodulatory activities of small host defense peptides. Antimicrob Agents Chemother.

[43] Scott MG, Vreugdenhil AC, Buurman WA, Hancock RE, Gold MR (2000). Cutting edge: cationic antimicrobial peptides block the binding of lipopolysaccharide (LPS) to LPS binding protein. J Immunol.

[44] Bosca L, Zeini M, Traves PG, Hortelano S (2005). Nitric oxide and cell viability in inflammatory cells: a role for NO in macrophage function and fate. Toxicology.

[45] Haq I, Chowdhry BZ, Jenkins TC (2001). Calorimetric techniques in the study of high-order DNA-drug interactions. Methods Enzymol.

[46] Roy A, Kucukural A, Zhang Y (2010). I-TASSER: a unified platform for automated protein structure and function prediction. Nat Protoc.

[47] Yang J (2015). The I-TASSER Suite: protein structure and function prediction. Nat Methods.

[48] Zhang Y (2008). I-TASSER server for protein 3D structure prediction. BMC Bioinformatics.

[49] Landers TF, Cohen B, Wittum TE, Larson EL (2012). A review of antibiotic use in food animals: perspective, policy, and potential. Public Health Rep.

[50] Henzler Wildman KA, Lee DK, Ramamoorthy A (2003). Mechanism of lipid bilayer disruption by the human antimicrobial peptide, LL-37. Biochemistry.

[51] Sochacki KA, Barns KJ, Bucki R, Weisshaar JC (2011). Real-time attack on single Escherichia coli cells by the human antimicrobial peptide LL-37. Proc Natl Acad Sci USA.

[52] Volgers C, Savelkoul PHM, Stassen FRM (2018). Gram-negative bacterial membrane vesicle release in response to the host-environment: different threats, same trick?. Crit Rev Microbiol.

[53] Suzuki K (2016). Human Host Defense Cathelicidin Peptide LL-37 Enhances the Lipopolysaccharide Uptake by Liver Sinusoidal Endothelial Cells without Cell Activation. J Immunol.

[54] Mookherjee N (2006). Modulation of the TLR-mediated inflammatory response by the endogenous human host defense peptide LL-37. J Immunol.

[55] Dathe M, Wieprecht T (1999). Structural features of helical antimicrobial peptides: their potential to modulate activity on model membranes and biological cells. Biochim Biophys Acta.

[56] Beevers AJ, Dixon AM (2010). Helical membrane peptides to modulate cell function. Chem Soc Rev.

[57] Dathe M, Nikolenko H, Meyer J, Beyermann M, Bienert M (2001). Optimization of the antimicrobial activity of magainin peptides by modification of charge. FEBS Lett.

