# Supplementary material for: Cathelicidins PMAP-36, LL-37 and CATH-2 are similar peptides with different modes of action
Source: Sci Rep. 2019 Mar 18;9:4780. doi: 10.1038/s41598-019-41246-6 (PMC6423055; doi:10.1038/s41598-019-41246-6)
Supplement: Supplementary file 1 — Supplementary figures [file 41598_2019_41246_MOESM1_ESM.pdf]

Supplementary files

Cathelicidins PMAP-36, LL-37 and CATH-2 are similar peptides with different modes of action

Maaike R. Scheenstra,[1,#] Matthias van den Belt, Johanna L.M. Tjeerdsma-van Bokhoven, Viktoria A.F. Schneider, Soledad R. Ordonez, Albert van Dijk, Edwin J.A. Veldhuizen, Henk P. Haagsman.

[1]Department of Infectious Diseases and Immunology, Division of Molecular Host Defence, Faculty of Veterinary Medicine, Utrecht University, Utrecht, The Netherlands

Footnotes

[#]To whom correspondence may be addressed. E-mail: m.r.scheenstra@uu.nl. Telephone: +31302531141.

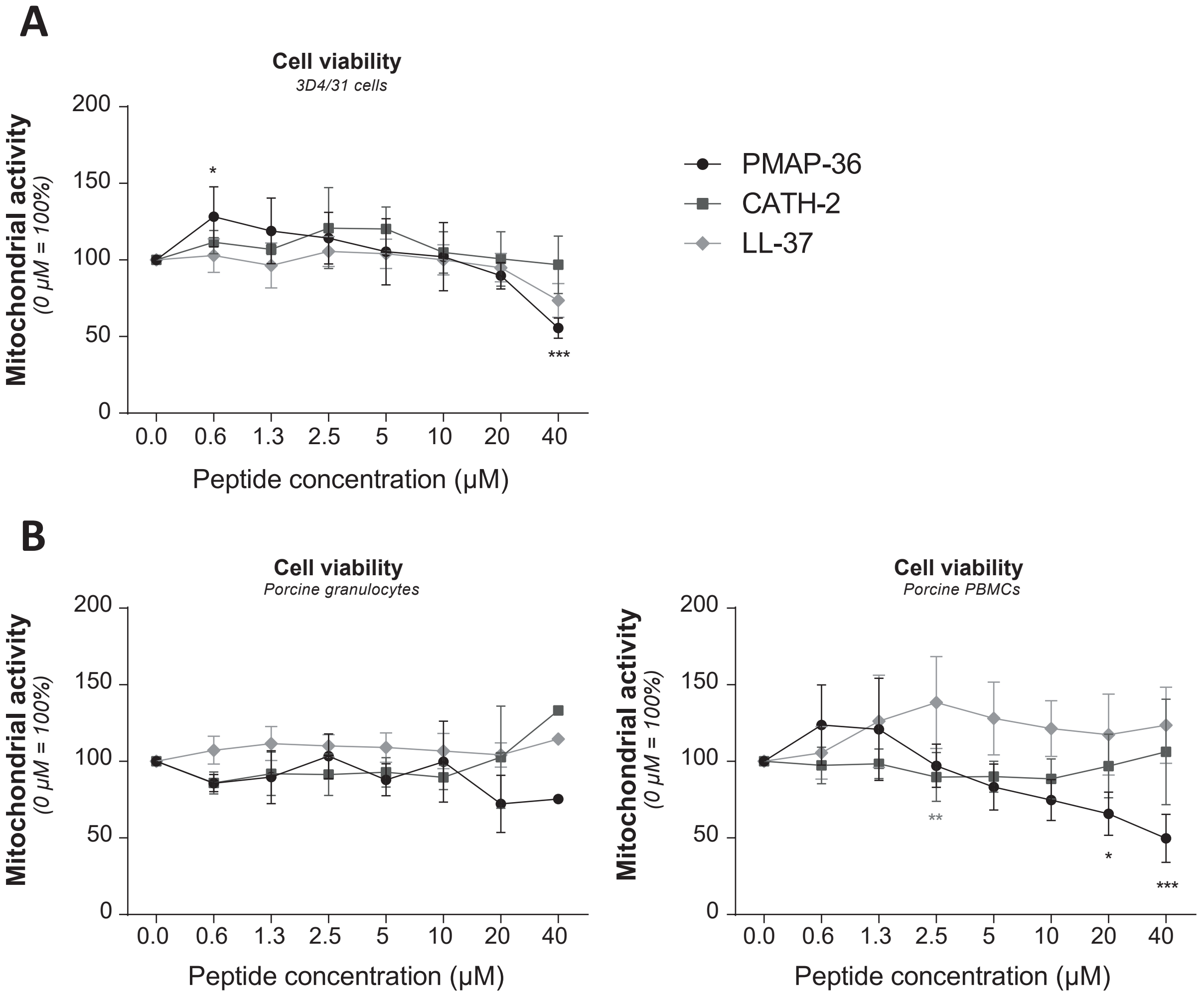

Supplementary Figure 1. Cytotoxic effect of PMAP-36, CATH-2 and LL-37 against a porcine cell line and primary cells

Cytotoxic effects against a porcine macrophage-like cell line, 3D4/31 cells, were tested using a WST-1 assay, indicating cell viability. No peptide control was set to 100% cell viability (A). Cytotoxic effects of PMAP-36, CATH-2 and LL-37 against freshly isolated primary cells. Granulocytes and PBMCs were isolated from blood, using Ficoll-Paque Plus separation and red blood cell lysis (B). Data is plotted as average +/- s.d. (3D4/31 cells N=4, granulocytes N=3, PBMCs N=6). Samples were compared to the no peptide control, using two-way ANOVA with the Bonferroni post-hoc test. (*=p≤0.05; **=p≤0.01; ***=p≤0.001;****=p≤0.0001; black - PMAP-36; dark gray – CATH-2; light gray – LL-37)

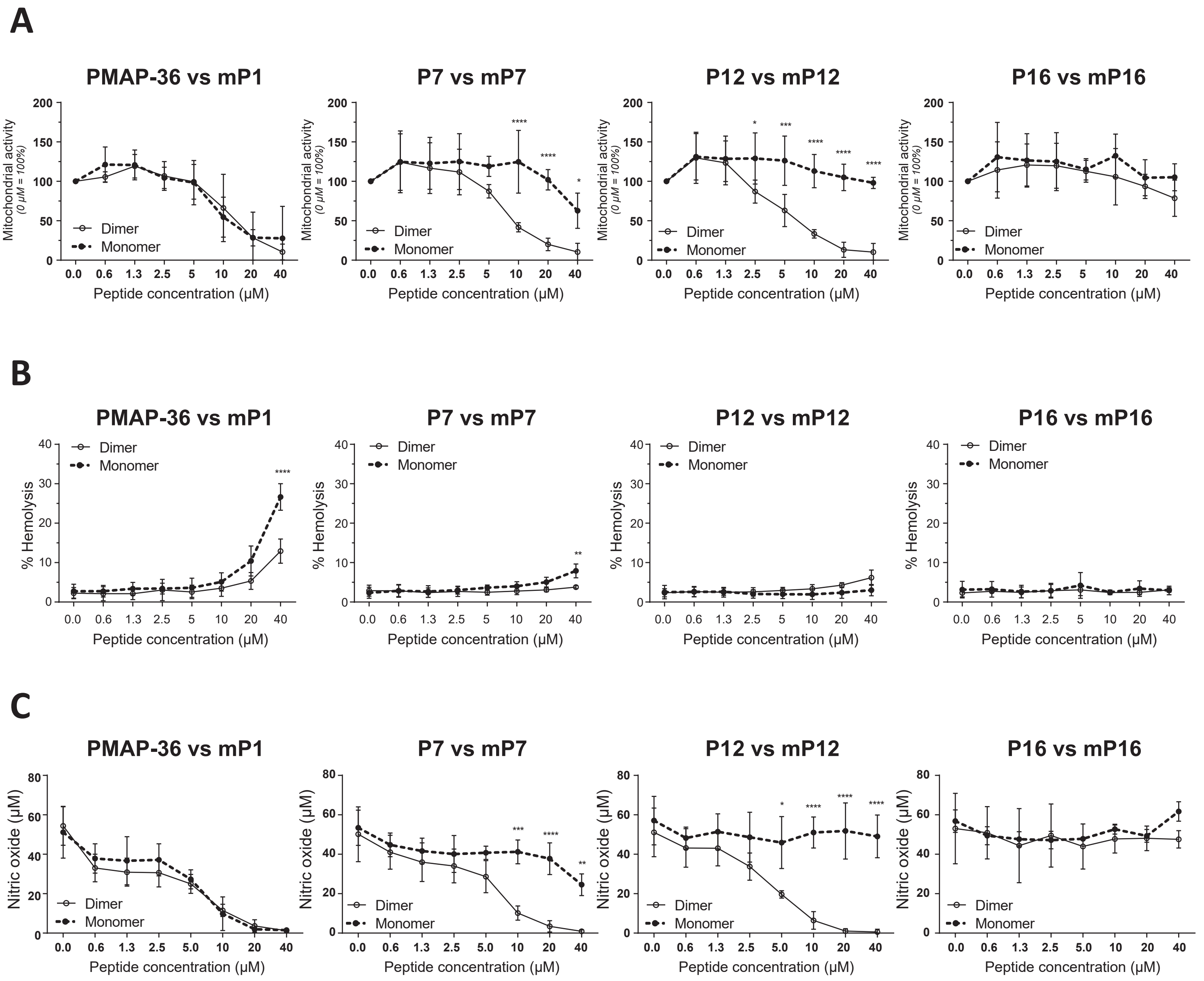

Supplementary Figure 2. Cytotoxic, hemolytic and LPS neutralizing and binding capacity of PMAP-36 monomeric analogs Cytotoxic effects against RAW264.7 cells were tested using a WST-1 assay, indicating cell viability. No peptide control was set to 100% cell viability (A). Hemolytic effect on porcine red blood cells was determined by heme release. The positive control, 0.2% Triton was set to 100% lysis and no peptide control as background lysis (B). The PMAP-36 analogs were tested for their ability to neutralize LPS O111:B4. NO production by RAW264.7 cells is depicted (C). Data is plotted as average +/- s.d. with N=3. Results of monomeric peptides were compared to its dimeric equivalent peptides (molarity calculated for monomer peptides), using two-way ANOVA with the Bonferroni post-hoc test. (*=p≤0.05; **=p≤0.01; ***=p≤0.001;****=p≤0.0001)
